# The American Veterinary College

**Published:** 1889-01

**Authors:** 


### Art. VIII—THE AMERICAN VETERINARY COLLEGE.

The year 1875 will always be considered an eventful one in the history of Veterinary Medicine in the United States.

After eleven years of hard and faithful work in assisting in the foundation of a Veterinary College, the entire faculty of that dying institution were obliged to resign in a body, and to cancel their connection with the chartered organization which they had literally speaking nursed and raised ; but if they separated from their original seat of labors they did not become blind to the importance of their calling, they did not ignore the necessity nor the value of a continuation of their work, and it was soon decided to make all necessary efforts to proceed with the work again, resuming their labors so suddenly and unexpectedly suspended. It was then that the organization of the American Veterinary College was effected. This important event took place April 1875, when a few gentlemen, friends of the faculty, availed themselves of a general law of the State of New York, and having complied with all its requirements issued their first announcement. The faculty consisted of Dr. A. Liautard, V. M., Prof. of Comparative Anatomy and Operative Surgery; Dr. A. Large, M. R. C. V. S. Prof. of Veterinary Practice and Clinical Medicine; Dr. F. D. Weisse, Lecturer on General Pathology ; Dr. A. W. Stein, Prof.

of Comparative Physiology and Histology ; Dr. S. R. Percy, Prof. of Materia Medica and Therapeutics ; Dr. J. L. Robertson, D. V. S., Prof. of Cattle Pathology and Obstetrics ; Dr. F. Leroy Satterlee, Prof. of Chemistry.

Few changes took place in the organization of this teaching body for several years, but it was gradually modified until about seven years ago, when the changes and additions that were made brought it to what it is to-day one of the most complete teaching bodies in the country.

In the fourth year of the existence of the American Veterinary College the Board of Regents of the University of the State, upon the report made by one of its members, Mr. E. C. Benedict, granted it a recognition heretofore denied to all other institutions of this kind, and the result of this official recognition was a large increase in the number of students, which necessitated a change in the teaching facilities of the College. The lecture room was then considerably enlarged, the dissecting room improved and material for clinical studies increased. In the tenth year an important event took place in the History of the Veterinary Profession in New York, viz : The consolidation of the Veterinary Colleges, then in existence in New York City, the Columbia Veterinary College transferring to the American Veterinary College all the rights, franchises and clientage. The act of incorporation under which the American College had so far carried on its labors was however, through peculiar circumstances, proving detrimental to its professional standing and with a view of establishing itself on a basis which would be satisfactory to all, the Board of Trustees, obtained in 1886 a special Act of the Legislature sanctioning the original organization, legalizing all the work previously performed, and placing the College on an equal footing with that of any other institution of learning. Success was assured. The students increased in number, and it was found necessary to add another building, using one for college and clinical purposes and the other as a hospital. This was accomplished in the session of 1886 and 1887, and to-day these two buildings offer students all the facilities that can be desired. The increase in the number of students of this institution is shown in the report of its thirteenth year, which states that the class numbered eighteen, the first year of its organization, and 132 in the session of 87-88. Its Alumni Association counts to-day 268 graduates. The theory and the practice of teaching advance together. The clinical opportunities are very great. Over 30,000 animals during the last thirteen years have been brought before the students, several thousand surgical operations having been witnessed by them. Since the foundation great changes have been made in the course of lectures and length of studies of this College, and additions have been made yearly as the need was felt in the various branches of Veterinary education. The governing faculty has to-day seven professors, all of which are graduates of Human Medecine, four of them holding Veterinary degrees obtained in various Colleges abroad, and at home. The Adjunct faculty has ten lecturers, all but two of which are Veterinarians, and while the special branches of Anatomy, Surgery, Equine, Cattle and Canine Pathology, Physiology, Chemistry, Obstetrics, Materia Medica and Therapeutics are treated by the governing faculty, lectures on Opthalmology, Histology, Horseshoeing, Surgical Pathology, Jurisprudence and Sanitary Medicine, are trusted to the care of the Adjunct faculty. Special teaching is given on Operative Surgery, Practical Microscopy, Toxicology and Chemical Analysis.

Such is briefly the history of the American Veterinary College, which claims the title of the oldest school in the United States in permanent active work and as a teaching body of nearly twenty-five years experience. Her life has been a series of successful progress, and yet there is something which may endanger her existence. If this is serious danger for the American Veterinary College, it is no less so, however, for all the other American Veterinary Schools except the Veterinary Department of the University of Pennsylvania. This has been appreciated by the officers who have for years managed its affairs. The American Veterinary College is as yet, like the Canadian Schools, and others in the United States, *but a private undertaking*. Unlike the University of Pennsylvania it has had no outside assistance. Truly its Alumni united generously to create a fund to secure land as the site for the erection of a suitable building. An entirely new Act of the Legislature authorizing the reorganization of the College and granting her special privileges of exceptional nature has been obtained. Attempts have been made to arouse in the minds of the citizens of New York, generous feelings which will prompt them to assist pecuniarily in the work that the Trustees and faculty have been doing, and yet, notwithstanding all this, the

American Veterinary College though it has made for itself a world-wide reputation, and though she can claim without fear the name of Alma Mater of the American Veterinary profession, is yet under a serious disadvantage which will continue until it is established under the roof of a building the character of which will not be of the private nature held since the organization of the college.

